# Infrared thermography fails to visualize stimulation-induced meridian-like structures

**DOI:** 10.1186/1475-925X-4-38

**Published:** 2005-06-15

**Authors:** Gerhard Litscher

**Affiliations:** 1 Biomedical Engineering and Research in Anesthesia and Intensive Care Medicine, Medical University of Graz, Austria

## Abstract

**Background:**

According to Traditional Chinese Medicine (TCM) the vital energy flows through a system of channels also called meridians. Generally accepted proof for meridians cannot be considered as being given. Goal of this study was to examine whether possible stimulation-induced meridian-like structures, as recently described by other authors, can be visualized and objectified simultaneously at different infrared wavelength ranges.

**Methods:**

The study analyses evidence for the existence of acupuncture-specific, meridian-like artifacts in 6 healthy volunteers (mean age ± SD 28.7 ± 3.7 years; range 25 – 35 years). Two infrared cameras at different wavelength ranges were used for thermographic control of possible stimulation effects (moxibustion-cigar, infrared warmth stimulation, needle and laserneedle stimulation). In addition to thermography, temperature and microcirculatory parameters were registered at a selected point using laser-Doppler flowmetry.

**Results and Conclusion:**

After moxibustion (or infrared light stimulation) of the body at 2 – 5 μm and 7.5 – 13 μm ranges, different structures appear on thermographic images of the human body which are technical artifacts and which are not identical to what are known as meridians in all textbooks of TCM. Further scientific studies are required regarding the possible visualization of meridians.

## I. Background

Acupuncture is an essential component of Traditional Chinese Medicine and is finding increased application in therapeutic concepts for treating various illnesses. The most important effective structural elements are acupoints which are divided topographically into specific meridians. Many references regarding the qualitative characteristics of acupoints are available and histological tests seem to prove this for a number of acupoints [1]. Attempts to visualize the course of "energetic paths" in acupuncture using physical-technical methods, are described by different authors. However, the results from investigations available up until now are controversial and generally accepted proof for meridians cannot be considered as being given [2-5].

One goal of this study was to examine whether possible stimulation-induced meridian-like structures, as recently described by other authors [2], can be visualized and objectified using modern biomedical engineering methods of thermography [6], simultaneously at different infrared wavelength ranges.

## II. Methods

### A. Thermography

Two infrared cameras at different wavelength ranges were used for thermographic control of possible stimulation effects.

The Agema Thermovision™ 470 PRO infrared camera system with optics of 20° (Flir Systems Inc., Portland, USA) operates at a wavelength range from 2 – 5 μm. The temperature measurement range lies between -20°C and +500°C and can be increased to 2000°C using filters. Sensitivity is 0.1°C at 30°C. The system is ready for use in 15 – 20 seconds.

A second infrared camera was used simultaneously with the system above during all measurements. Here, a FLIR ThermaCAM™ S65 system with 24° optics was used. The spectral range of this camera lies between 7.5 and 13 μm and the temperature range lies between -40°C and 1500°C (2000°C optional). Temperature differences of less than 0.08°C (30°C, 50 Hz) can be registered with this camera. Using a special detector (FPA) with 320 × 240 pixel geometric resolution of 76.800 pixel per picture can be achieved.

The determined data was transferred to a Notebook using ThermaCAM QuickView™ software and analysed with ThermaCAM Researcher Pro 2.8 software (Flir Systems Inc., Portland, USA).

### B. Laser-Doppler-flowmetry

**Figure 4:**
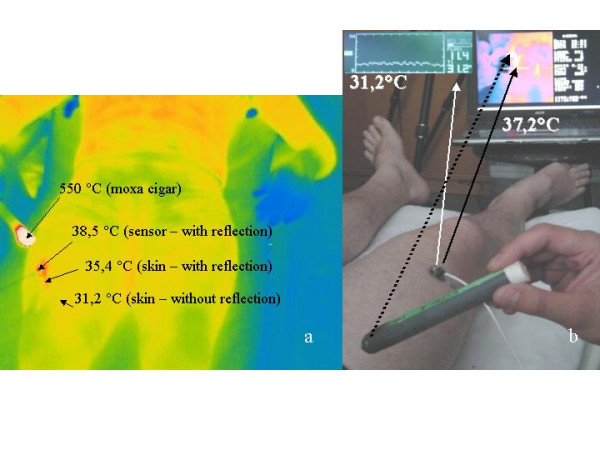
Experimental set-up to demonstrate elevated false temperature values in the thermograms (left 2 – 5 μm; right above 7.5 – 13 μm) predominantly caused by reflection. In the thermogram, the actual surface skin temperature is noted as 31.2°C measured at a point next to the reflection using additional temperature sensor equipment (above center).

In addition to thermography, temperature and microcirculatory parameter flux ( = product of average speed and concentration of moving red blood cells in the tissue sample volume) at the selected point on the thigh (compare Fig. 4), were registered as comparative measurements using a Laser-Doppler device (DRT4) from Moor Instruments Ltd. (Devon, England). Laser wavelengths were 780 nm, whereby the raw signal was filtered with a digital filter from 20 Hz to 22.5 kHz.

### C. Stimulation methods

#### a. Moxibustion-cigar and infrared warmth stimulation

A burning moxibustion-cigar made of mugwort (length 20 cm, diameter 1.5 cm, Hunan, China) was used for stimulation. This radiates an average temperature of 550°C (measured with FLIR ThermaCAM S65) with an energy maximum ranging from 3 to 3.5 μm [7].

The second stimulation method (infrared heat radiation) was performed with an infrared lamp (EWT, 150 W, Infrared R95E, Philips, Eindhoven, Netherlands).

#### b. Needle- and laserneedle stimulation

In addition to the first two stimulation methods without actual skin contact, a further standardized acupuncture scheme was used, from which we know it induces proven, reproducible effects in the brain [8-10]. Following acupoints were stimulated with manual needle and laserneedle acupuncture: Neiguan (Pe.6), Qihai (Ren 6), Zusanli (St.36) and Sanyinjiao (Sp.6) [8]. Additional moxibustion was performed at acupoint Qihai [8].

The laserneedles used emitted continuous laser light at a wavelength of 685 nm and output of 30 – 40 mW per laserneedle. Stimulation time was 10 minutes resulting in an energy density of 2.3 kJ/cm^2 ^at each laserneedle and an average total value of 9.2 kJ/cm^2 ^for the entire duration of laser stimulation [11-16]. Identical acupoints as by needle acupuncture were used. No moxibustion was done during this type of stimulation.

### D. Volunteers

A total of 6 healthy volunteers were examined. Two volunteers were women and 4 were men, mean age ± SD was 28.7 ± 3.7 years (range: 25 to 35 years). Average body height was 176.2 ± 11.0 cm and the average body weight was 83.7 ± 23.8 kg. Volunteers declared that they did not take any medication. The study was approved by the Ethics Committee of the Medical University of Graz (13-048) and all participants gave their written consent.

### E. Procedure

**Figure 1:**
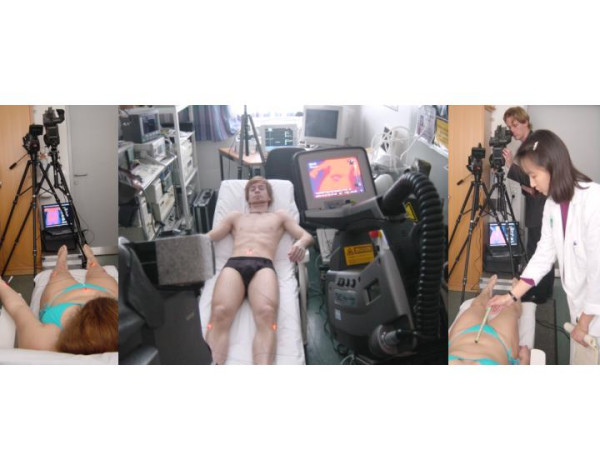
Infrared analytical measurement set-up (center), stimulation with moxa-cigar (right) and stimulation with laserneedle acupuncture (left) in the biomedical laboratory at the Medical University of Graz.

A standardized study design was used on all volunteers and included the following steps:

The volunteers were laid relaxed on a bed for 10 minutes before the examination was started. During this time, the required measurement devices were applied (Fig. 1). The first stimulation method was performed with a moxa-cigar. Different areas of the body (left leg, right leg, upper body) were stimulated at a distance of about 10 cm for about 5 minutes. After a resting period, the procedure was repeated using the infrared lamp. In the third part of this study design a standardized acupuncture scheme was used and stimulation with laserneedles, manual needle acupuncture and additional moxibustion of the acupoint Qihai for a duration of 10 minutes was performed. The order of stimulation was selected at random [9].

## III. Results

We could not visualize structures which could be connected with possible meridians, as described in Traditional Chinese Medicine, in any of our volunteers. We were able to clearly objectify and quantify technical reflection artifacts; however, indications for a biological correlation could not be found (Tab. 1). In the cases when only needle or laserneedle (685 nm) stimulation was used no thermal reflection phenomena were present and also no meridians could be detected (compare Tab. 1).

**Table 1:** Meridian structures, as described in Traditional Chinese Medicine, could not be proven in any of the volunteers. However, artifacts were evident in all volunteers.

**Visualization**							
	reflection phenomena (artifacts)			meridian structures			

volunteer #	moxa-cigar	infrared warmth stimulation	arbitrary meridian-structures	moxa-cigar	infrared warmth stimulation	needle acupuncture	laserneedle acupuncture

1	+	+	+	-	-	n.	-
2	+	+	+	-	-	n.	-
3	+	+	+	-	-	n.	-
4	+	+	+	-	-	n.	-
5	+	+	+	-	-	-	n.
6	+	+	+	-	-	-	n.

+ ... detectable; – ... not detectable; n. ... not performed

**Figure 2:**
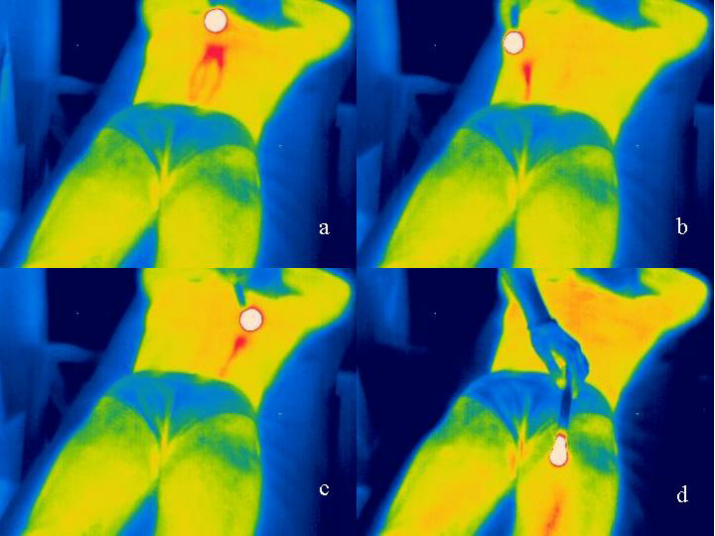
Thermograms from a 30-year-old volunteer with reflection artifacts. The moxa-cigar is visible as a circular white area. Dependent on the angle of reflection, technical reflection effects visible as lines at optional parts of the body (a – d) can occur. The thermographic scale was defined from blue (25°C) to red/white (41°C).

Figure 2 shows the reflection (mirror-like) artifacts which result from reflection and scattering, in a 30-year-old volunteer. According to the position of the moxa-cigar (circular white area) in dependency with the camera position, reflections showing an optical linear path on the thermogram, can be applied to optional areas of the body.

**Figure 3:**
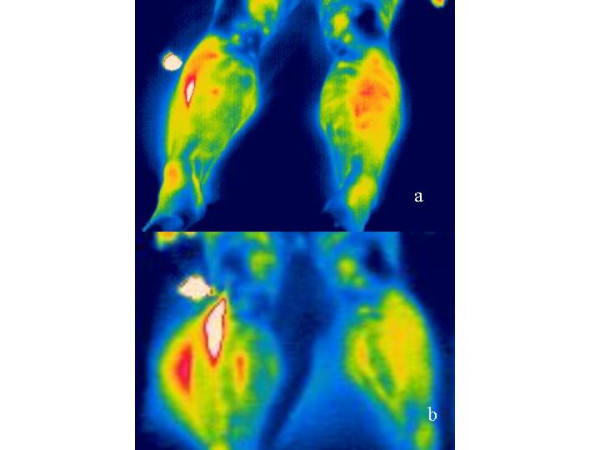
Simultaneous monitoring of reflection artifacts with an infrared camera at a 7.5 – 13 μm (a) and a camera at a 2 – 5 μm (b) wavelength range. Note the stronger expression of the artifact (white-red area on the foot) at a wavelength of 2 – 5 μm. The moxa-cigar is shown as the white circular area next to the leg. For further details see Fig. 2.

In this manner, typical artifacts from each volunteer can be determined in the "thermographic pictures". Figure 3 shows a further example for this phenomenon. Here, simultaneous measurements in two different wavelength ranges were made using both infrared cameras. While the first camera (7.5 – 13 μm; Fig. 3a) yielded less reflection artifacts with an all over better optical resolution, the second camera (2 – 5 μm) revealed more distinct artifacts. The energy maximum of the moxa-cigar lies in the range of 3 – 3.5 μm. Since the radiation curve markedly flattens at 7.5 – 13 μm obviously greater reflection occurs within the range of 2 – 5 μm.

Figure 4 demonstrates the occurrence of reflection phenomena in a 35-year-old volunteer at the thigh. Simultaneously, surface skin temperature and flux were determined with a combined temperature and microcirculatory sensor which was applied to the skin. The reflection artifacts at the area of the sensor led to false temperature representation of 38.5°C at the sensor surface (smooth plastic surface) in the thermograms from both cameras. The neighboring skin surface revealed a 4.2°C higher temperature (35.4°C instead of 31.2°C) in the thermograms. The taped on temperature sensor confirmed the correct value of 31.2°C for surface skin temperature at this point.

**Figure 5:**
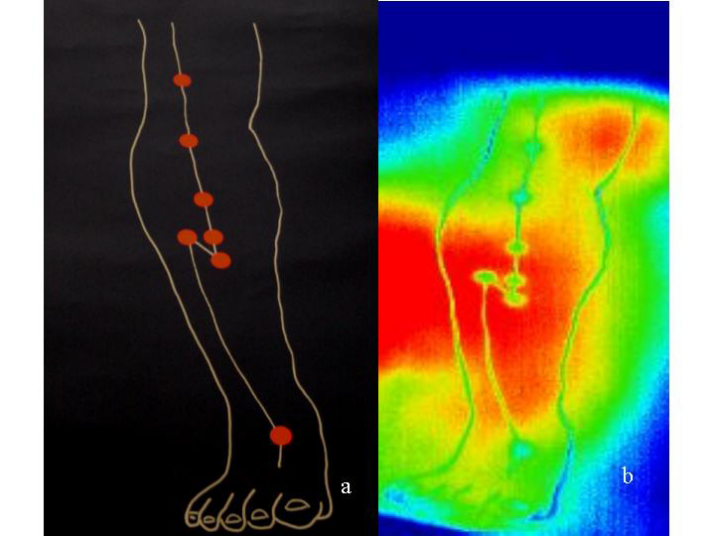
Visualization of marked paths and points on a black carton (a) in the thermogram (b). Note that this visualization was created by a stimulation with a heat source at a distance of 10 cm.

Figure 5 shows the structures which were registered on a black carton (Fig. 5 left). Due to the optical characteristics in the infrared range, the marked (linear type) structure after external stimulation with an infrared heat source is visible in the thermogram.

## IV. Discussion

Thermic and infrared energy lies within a wavelength range no longer detectable by the human eye. This energy lies within the region of the electro-magnetic spectrum which is perceived as being warm. Contrary to visible light, every object in this region with a temperature over absolute zero radiates heat. When the temperature of an object is higher, the intensity of emitted infrared radiation is also higher. Infrared cameras produce pictures of invisible infrared- or heat radiation, thus enable exact temperature measurements.

This technique of temperature measurement has already been used in several studies dealing with acupuncture research [2-6]. Advantages of this method are the high local resolution as well as optical data registration without requiring skin contact. In addition, the method is passive, i.e. no energy is transmitted into the body and thus is completely harmless. This allows longer examination times and therefore can also be used on infants [17].

Dynamic changes in temperature distribution, as possible during acupuncture can be registered well and reliably [6]. Areas which produce less heat or are less perfused are shown as much cooler on the calculated pictures as those with better circulation. Differences in temperature which are less than 0.08°C can be objectified. An important factor is the emission coefficient of the skin, which lies at 0.97 in this study. This factor is dependent upon the direction of radiation. For this reason, border regions are visible as being much cooler. The misrepresentation of the measurement value resulting from geometrics of the radiated structures must be considered during interpretation [7]. Errors can also occur because of false emission coefficients at the skin because e.g. greasy skin creams can lead to an obvious reduction in the emission coefficient [6].

Our experience shows that material characteristics such as reflection or refraction, two important examples in this study, are dependent upon the wavelength of optical radiation, and can show strong differences in the visible spectral range. Thermal radiation spreads straight out in a homogenous medium and is influenced by border areas. For example, when air borders on a smooth metal surface, reflection occurs. An example for directed reflection is the surface mirror. Ideal diffuse reflection can be achieved with bright white finishes and coarse surfaces. On most border surfaces, mixed types of reflection occur. Further, part of the radiation is absorbed by the surface.

Reflection is very important, particularly in thermography. In the majority reflection is unwanted in the radiation path of optical instruments and should be minimized. Often, background rays mask the measurement signal due to the reflection in irregular surfaces of the measurement object including the human body. This effect partially determines the limited applicability of infrared cameras. It is important to note, that the optical laws are valid for the entire spectral range; obvious deviations are evident at the borders of extreme ultraviolet or extreme infrared radiation [7].

The technical, but obviously not biological reflection phenomena described in this study can be applied to any desired area of the body. "Thermic pictures" with partially well known characteristics in the temperature range between 20°C and 40°C above the filmed body region can be made. The fact that we are here dealing with technical artifacts and not biological phenomena can be justified by the following:

1. Reflections can be applied to optional parts of the body depending on the position of the moxa-cigar in connection with the angle of incidence of both cameras. In addition, the structure and form of phenomena (circular, linear) in dependence with the values described above can be varied. Thus, correspondence with the meridian structures according to Traditional Chinese Medicine is not given (compare Fig. 2,3,4).

2. The moxa-cigar radiates more than 550°C with maximum energy ranging between 3 and 3.5 μm. The radiation curve flattens markedly between 7.5 and 13 μm, thus, stronger reflections in the range of 2 – 5 μm occur than in the long-wave infrared range. This could be illustrated for the first time using two infrared cameras (compare Fig. 3).

3. Reflection phenomena (artifacts) are also reproducible in both wavelength ranges in non-vital structures such as bed linens or paper (compare Fig. 5).

4. Simultaneous measurement of surface temperature and microcirculation did not show any changes in regard to temperature and circulation. On the contrary, temperature differences up to 7°C could be registered between both methods at the examined regions of the body which underlines the probability of an artifact (compare Fig. 4).

5. So-called moxa-induced meridian paths can be projected (reflected) horizontally or diagonally at any angle (compare Fig. 2).

6. Stronger reflections at both wavelength ranges (2 – 5 μm and 7.5 – 13 μm) can be triggered by using an infrared stimulation device.

7. No path equivalent meridians on the body surface could be visualized in any volunteer during acupuncture stimulation (manual needle acupuncture and laserneedle stimulation as well as moxibustion).

## V. Conclusion

In conclusion, we note that thermographic methods such as infrared cameras at wavelength ranges of 2 – 5 μm and 7.5 – 13 μm and other High-Tech methods [8-16,18-25] are effective complementary methods in acupuncture research which support demystification of this treatment method. However, the validity of the method for proving meridian structures according to the view of Traditional Chinese Medicine, must be considered critically and analysed scientifically. In a publication which attempted to visualize meridians and their path using infrared thermography, the authors [2] described that the left stomach meridian could be visualized during stimulation with a moxa-cigar near the left foot. At the same time, the right spleen meridian should be visualized on the right foot. Based on the foundations of Traditional Chinese Medicine, it is improbable that exactly these two meridians can be activated simultaneously. According to current technical standings, the visualization of energetic paths in the sense of meridians as described in recent literature is not possible using thermography. On the contrary, the supposed thermographic reproduction of meridians is dependent upon physical-technical artifacts caused by thermic reflections.

According to current technical standings and to the method proposed by other authors [2], the visualization of energetic paths in the sense of meridians seems to be not possible using thermography. Further scientific studies are required regarding the possible visualization of meridians.
